# Variant information systems for precision oncology

**DOI:** 10.1186/s12911-018-0665-z

**Published:** 2018-11-21

**Authors:** Johannes Starlinger, Steffen Pallarz, Jurica Ševa, Damian Rieke, Christine Sers, Ulrich Keilholz, Ulf Leser

**Affiliations:** 1 Department of Computer Science, Humboldt-Universität zu Berlin, Unter den Linden 6, Berlin, 10099 Germany; 2 Charité Conprehensive Cancer Center, Charité Unviersitätsmedizin Berlin, Charitéplatz 1, Berlin, 10117 Germany; 3 Institute of Pathology Molecular Tumor Pathology, Charité Unviersitätsmedizin Berlin, Charitéplatz 1, Berlin, 10117 Germany; 4 Department of Hematology and Medical Oncology, Campus Benjamin Franklin, Charité Unviersitätsmedizin Berlin, Hindenburgdamm 30, Berlin, 12203 Germany; 5 Berlin Institute of Health (BIH), Kapelle-Ufer 2, Berlin, 10117 Germany; 6 Department of Anesthesiology and Operative Intensive Care Medicine (CCM/CVK), Charité Unviersitätsmedizin Berlin, Charitéplatz 1, Berlin, 10117 Germany

**Keywords:** Molecular cancer therapy, Variant information system, Data model, Genomic variant data integration

## Abstract

**Background:**

The decreasing cost of obtaining high-quality calls of genomic variants and the increasing availability of clinically relevant data on such variants are important drivers for personalized oncology. To allow rational genome-based decisions in diagnosis and treatment, clinicians need intuitive access to up-to-date and comprehensive variant information, encompassing, for instance, prevalence in populations and diseases, functional impact at the molecular level, associations to druggable targets, or results from clinical trials. In practice, collecting such comprehensive information on genomic variants is difficult since the underlying data is dispersed over a multitude of distributed, heterogeneous, sometimes conflicting, and quickly evolving data sources. To work efficiently, clinicians require powerful Variant Information Systems (VIS) which automatically collect and aggregate available evidences from such data sources without suppressing existing uncertainty.

**Methods:**

We address the most important cornerstones of modeling a VIS: We take from emerging community standards regarding the necessary breadth of variant information and procedures for their clinical assessment, long standing experience in implementing biomedical databases and information systems, our own clinical record of diagnosis and treatment of cancer patients based on molecular profiles, and extensive literature review to derive a set of design principles along which we develop a relational data model for variant level data. In addition, we characterize a number of public variant data sources, and describe a data integration pipeline to integrate their data into a VIS.

**Results:**

We provide a number of contributions that are fundamental to the design and implementation of a comprehensive, operational VIS. In particular, we (a) present a relational data model to accurately reflect data extracted from public databases relevant for clinical variant interpretation, (b) introduce a fault tolerant and performant integration pipeline for public variant data sources, and (c) offer recommendations regarding a number of intricate challenges encountered when integrating variant data for clincal interpretation.

**Conclusion:**

The analysis of requirements for representation of variant level data in an operational data model, together with the implementation-ready relational data model presented here, and the instructional description of methods to acquire comprehensive information to fill it, are an important step towards variant information systems for genomic medicine.

**Electronic supplementary material:**

The online version of this article (10.1186/s12911-018-0665-z) contains supplementary material, which is available to authorized users.

## Background

Personalized, genome-based therapy has become a promising tool in modern oncology. Its basis is the analysis of the mutational profile found in a patient’s tumor, i.e. its particular set of genomic variants, and our (limited) knowledge of how these mutations might influence tumor progression and individual druggability. To allow interpretation of a given mutational profile, oncologists require a concise, yet comprehensive set of information describing each detected variant. This includes the variant’s biological impact on (tumor) cell function, prevalence in different tumor types, ongoing, past or planned clinical trials targeting this variant, results from genome-wide association studies (GWAS) etc. Such information can only be obtained when consulting multiple, distributed, and heterogeneous databases, such as ClinVar [1], CIViC [2], COSMIC [3], DrugBank [4], or KEGG [5]. These databases have different updating strategies, use different identifiers and terminologies, and often focus on particular tumor types or therapy situations. As a consequence, finding the most relevant data and condensing it into a comprehensive overview on a given patient’s set of variants is difficult even for the expert. Today, this process of data integration is mostly done manually, with the molecular oncologist consulting a spectrum of at least half a dozen databases and additionally performing extensive literature search. Such manual, case-based search and data acquisition is enormously time consuming and prone to missing relevant data, and has been identified as one of the most pertinent bottleneck in precision medicine [6].

In recent work, requirements have been described regarding the necessary breadth and quality of variant-associated information to make them useful in the clinic [7–12]. These descriptions range from classification schemes and strategies for how the clinical relevance of a given variant may be quantified, to definitions of which data elements need to be captured for each variant to allow such classification. Such standards are of great importance for setting a common ground for variant level interpretation in clinical oncology. They do, however, only specify requirements, leaving aside the technical details of an actual implementation of such standards.

**Fig. 1:**
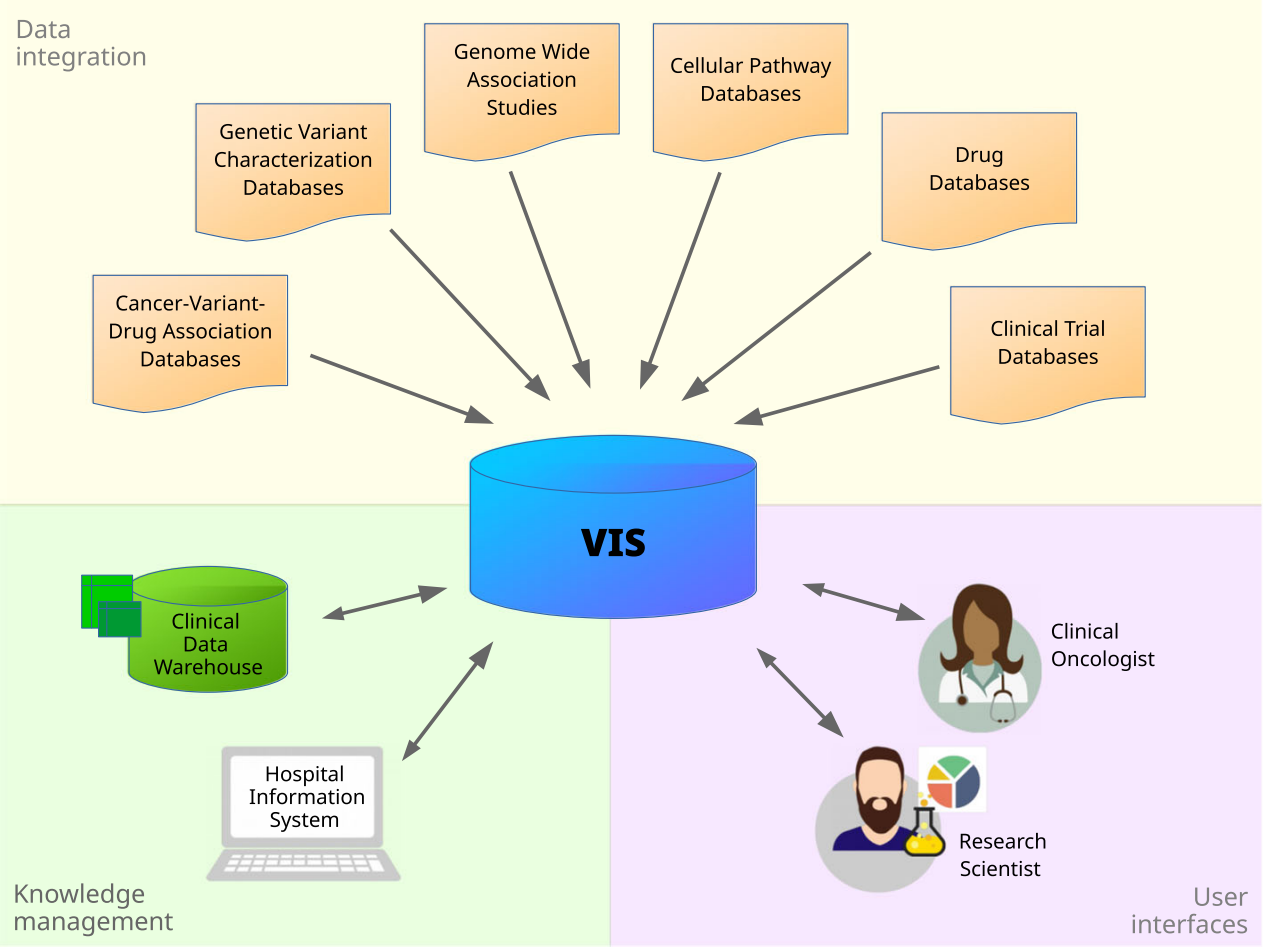
A Variant Information System (VIS) integrates public data sources and makes their joint information available for use both within inhouse systems for patient knowledge management and directly to domain expert users. (Clipart source: openclipart.org; public domain)

A Variant Information System (VIS) (see Fig. 1) offers such an implementation of the clinical needs regarding information on variants and provides a single point of access to search and retrieve this information. To this end, it has to address several conceptual and technical challenges: A VIS inevitably has to integrate a host of different primary databases. It must accurately model the biological reality behind this information, carefully setting variants into their specific genomic and transcriptional context and discerning between different isoforms. In addition to variant-centric data, it should include information regarding functional characteristics of affected genes, like their role in different cellular pathways or their druggability by pharmacological substances. A VIS should support the usage of multiple different ways to name biological entities (e.g., genes or drugs), or medical concepts (e.g., diseases or tumor grading), reflecting the existing semantic heterogeneity between the primary databases it is fed from. It also must be able to represent conflicting data, because results from different studies often differ, original databases are updated at different frequencies, and concepts are often not consistently defined across these databases; for instance, even the definition of basal cell-cycle related pathways differs considerably between different pathway databases [13]. The necessity to cope with different reference genomes and different gene models in the source databases adds further complications. Eventually, a VIS must implement robust and flexible update mechanisms to achieve a high level of currency despite its quickly and independently evolving base systems [14].

Taking these considerations into account, in this paper, we discuss two fundamental issues of implementing a comprehensive VIS: We (a) present a relational data model to accurately represent data extracted from public databases relevant for clinical variant interpretation, and (b) report on the technical design of integration pipelines to fill such a model with actual data. We build on our extensive experience in working with genomic and variant level data, and on lessons learned from applying such data in clinical cancer genomics.

## Methods

The implementation of a VIS is usually based on a database schema, which is a structured and computer-usable model of the data it works upon. Careful design of such a data model for variant level data in cancer is crucial for accurate representation of the complex biological, (bio)technological, medical, and pharmacological interdependencies between the various entities involved. Furthermore, it is a prerequisite for efficient storage and retrieval of this data for clinical interpretation.

In this section, we first describe the design principles underlying the construction of our data model, followed by an overview of the design process we applied. We then give account of the methods used for identification and integration of appropriate data sources to populate the resulting model in a VIS.

### Data model design principles

The design process of the data model was guided by a set of design principles which we initially compiled based on our own experience, and refined in the initial steps of the process using literature review. With each of the design principles listed here we indicate how it is reflected in the resulting data model, which will be described in more detail in the next section.

**Representation of biological entities** Modelling of entities and their relationships in a VIS has to be consistent both with the information needs of the clinical oncologist and with the knowledge actually available for the respective entities. This includes accurate modeling of their biological and therapeutic relationships. For instance, the data model has to reflect the complex interplay of variant identification, description, naming, and genomic positioning [15], or the diverse effects drugs can have based not only on variants themselves but also on other factors including the tumor type: Conceptually, the central entity of a data model for representation of variant level data for cancer therapy has to be a *Cancer Variant* that represents the therapeutic inseparability of a genetic variant and a specific cancer type. Representing entities also includes the definition of a concise set of core data elements most relevant for oncological interpretation of variant data; values for these core elements are mandatory to ensure interpretability. Besides such core data elements, entities may be described by additional, optional attributes. For instance, the amino acid substitution caused by a base-level variant is indispensable for clinical interpretation, whereas knowing the chromosome the mutated gene is located on may be optional. Although such optional information is typically not considered during systematic scoring of variants, having it readily available for investigative inspection is a functionality often requested by practitioners. Consequently, our data model can host a variety of information about variants, cancer types and their biological context. At the core, however, it is designed around a concise set of data elements most relevant for clinical interpretation.

**Alignment with community standards** The data model of a VIS should be consistent with existing community standards as much as possible. Most relevant to our work is the Minimum Variant Level Data (MVLD) set recently suggested by the Somatic Cancer Working Group of the Clinical Genome Resource [16] (Ritter et al. [7]), which defines a concise set of data elements necessary for descriptive and interpretive assessment of variants in cancer therapy. This information should be considered as core elements of any VIS data model. Regarding variant assessment, Li et al. [8] provide guidelines for aggregating primary evidences into easy-to-use scores as a basis for clinical decision-making. The authors define a categorization of variants into four tiers ranging from variants with strong clinical significance (Tier I) to benign variants (Tier IV). These guidelines have been developed for somatic variant interpretation by the Association of Molecular Pathologists (AMP) to complement the guidelines of the American College of Medical Genetics and Genomics (ACMG) for germline variants. While the actual interpretation of variant data in a given clinical context has to be left to the clinical oncologist, any VIS model should support implementation of guidelines for variant assessment. Furthermore, next to relevant data and guidelines for variant assessment, important functionality required from an application in support of precision oncology has been identified by the community from the user interface perspective [17], which a VIS has to be able to support. This includes the need for solutions to challenges posed by the use of multiple different terminologies, the presence of conflicting data, and the distribution of knowledge over a multitude of databases.

Following this principle, the selection of data elements to include in the core data set of our data model tightly follows the standard defined by Ritter et al. Furthermore, it explicitly allows integration of interpretive guidelines, which we showcase by the example of the *Clinical Relevance Score* for each genomic variant in a specific cancer type.

**Support for multiple namespaces and terminologies** Many, if not all entities relevant to variant level oncology can be identified by numerous different internationally used identification systems and ontologies [18]. Genes, for instance, can be identified by their Entrez Gene ID [19], Ensembl ID [20], RefSeq ID [21], or the UniProt ID [22] assigned to one of the proteins they code. Comprehensive tracking of different identifiers for each individual entity is necessary to allow integration of data from different sources. Special care must be taken to associate names and entities at the right level of granularity, to discern (or not), for instance, a given gene, its transcripts, its mRNAs and the protein isoforms it codes for. Similarly, different terminologies (or ontologies) exist for defining medical concepts like diseases, phenotypes, or the function of genes and drugs. For instance, cancer types are listed as concepts in SNOMED-CT [23], ICD-10 [24], the Human Phenotype Ontology [25], and the Disease Ontology [26], to name just a few. Even for a single cancer type within a single terminology, multiple names may be given. For instance, ICD-10 (CM 2017 C18.9) lists the following synonyms for the concept *colon cancer*: *adenocarcinoma of the colon*, *cancer of the colon*, *carcinoma of colon*, *malignant tumor of the colon*, and *malignant neoplasm of the colon*. Source databases of variant level information in cancer typically use any one of these synonyms to describe the respective entity - without including the unambiguous ID of the concept itself.

For our VIS data model, it is thus not only important to decide on a standardized target ontology with a coverage and a level of detail suitable for describing the respective entity types, but also to include links which map within and between different terminologies.

**Representation of conflicting data** Any implementation of an integrative data model must take into account the current situation that information on variants is dispersed over a multitude of databases and publications and that it is often contradictory and incomplete. For instance, the evidence level reported for the effect of a given drug on a given variant and cancer type may vary significantly from data source to data source. Even for less complex, presumably basic biomedical facts, contradictions are not uncommon: a given pathway may contain a different set of genes in Reactome [27] than in KEGG [5], transcripts may have different positions in Ensemble [28] and RefSeq [21], and diseases are structured differently in HPO [25] and UMLS [29]. Any VIS must decide on how to cope with this situation: It can either (1) deliberately choose only one source of knowledge, (2) perform some form of expert curation to derive unambiguous statements despite heterogeneous evidences, or (3) choose to transparently represent such cases in its own model, essentially leaving the responsibility of deciding which information to trust to the user.

The data model and integration pipeline we propose here for implementing a VIS follows the third option. The rational is that in the process of clinical decision making, full transparency regarding available information should be warranted.

**Linking to original evidences** All available knowledge on entities stored in a VIS must be backed by scientific evidence to allow evaluation of its trustworthiness for oncological decision making. The possibility of following data lineage from a stored fact back to its original evidence is especially important when gathering data from different data sources [30] - and even more when this leads to contradicting data as outlined above. Judging the quality and reliability of each information source in the face of heterogeneous and potentially contradicting data can not be done automatically.

Accounting for this issue, our data model is designed to not only capture heterogeneous information and to have it available for expert judgment, but to also associate each piece of information with a link to its original data source.

**Extensibility** In a field evolving as quickly as precision oncology, extensibility of a data model has to be a core aspect of the design process. For instance, while MVLD only addresses mutations on the genomic level, aberrations on the transcript level are starting to become increasingly important [11]. MVLD also captures information on single variants only, whereas research increasingly studies the impact of variant combinations or even mutational signatures [31]. Note that resolutions of these two issues are actually contained in the model presented here; however, the field of molecular oncology is evolving at a pace that rapidly changes and extends the underlying biological model itself. For instance, we decided – for now – to model only variants in coding regions, although it is quite likely that future clinical research will also include variants in non-coding regions. To be future-proof, a data model has to be able to easily accommodate additions and updates.

As a consequence, we not only include the aforementioned conceptual extensions to MVLD, but especially provide clearly defined extension mechanisms to guide further development of the model even in ways currently not foreseeable.

### Data model design process

Based on these design principles, our model was generated by an interdisciplinary team of computer scientists, clinical oncologists, and bioinformaticians. The design process followed an iterative approach, intermixing two complementary steps:

**1. Top-down** We studied several emerging standards for variant level data representation and interpretation to include a comprehensive range of elements and perspectives (e.g., [1, 2, 7–12, 17]). We also build on our own long-standing experience in working with data from the area of systems biology and systems medicine. This includes both technical expertise in integration of such data and its storage in relational databases and information systems (e.g., [32–35]), and practical application of such data in molecular oncology - and the insight of which information our own treatment decisions are influenced by.

**2. Bottom-up** From the other end, we studied several existing databases of variant level data (e.g., [1–4, 27, 36]). We mapped the data available in these databases to our expectations and experience of which data is relevant. Iteratively refining the model, we included additional data elements where necessary. From the start of the design process, it was clear that not all possible elements, data types, and tools could be included in the final model. Where necessary, we made an educated choice of which tools, ontologies, and data elements to include based on our own experience and literature review. However, we did construct the data model in an extensible way, as outlined in our design principles above, allowing these choices to be easily revised by the respective user.

### Data acquisition methodology

Existing public structured databases provide curated data that partially overlaps with the variant level data set described in our model. Several of these databases have to be integrated to acquire comprehensive variant level data covering all elements of the data model. This integration of data from source databases encompasses a) the selection and characterization of data sources to include and b) the setup of a technical integration pipeline for the integration process.

**Identification of source databases** From the multitude of databases available that provide data on genomic variants and their known relationships to cancer types, drugs, or both, we made a selection based on our experience of using these databases for manual aggregation of data for clinical decision making, and based on their respective coverage of elements relevant for our model.

As such, we characterized some of the most important public data sources (see Table 3 in *Results*), based on practical experience, third party reviews (e.g., [2, 8, 9]), and additional literature search (e.g, [37, 38]). In particular, we analyzed the selected databases with respect to the volatility of the information contained in them, the overlap in the data models employed by those databases, the terminologies used to identify respective entities, and potential conflicts and mappings between those terminologies.

**Integration of source databases** Taking from this characterization, we propose the design of a multi-step integration pipeline. This pipeline is based on well-established techniques of data integration [39] and assembles these to generate a materialized representation of the source data within the VIS. The rational for choosing a materialization as an endpoint of integration (as opposed to virtual integration with online acquisition of the source databases) lies in (a) increased fault tolerance, e.g., against failure of single sources in the assembly, (b) privacy regarding patient data, i.e., potentially identifying mutation profiles do not have to be transmitted to source databases at query time, and (c) better performance in query execution.

## Results

We here introduce our data model for variant level data for precision oncology, based on the design principles described in the previous section. We also present an integration process for filling such a model with facts extracted from public reference databases, such as COSMIC, ClinVar, or CIViC. We highlight areas that require special focus, such as methods for deriving aggregated risk scores.

### Data model

**Fig. 2:**
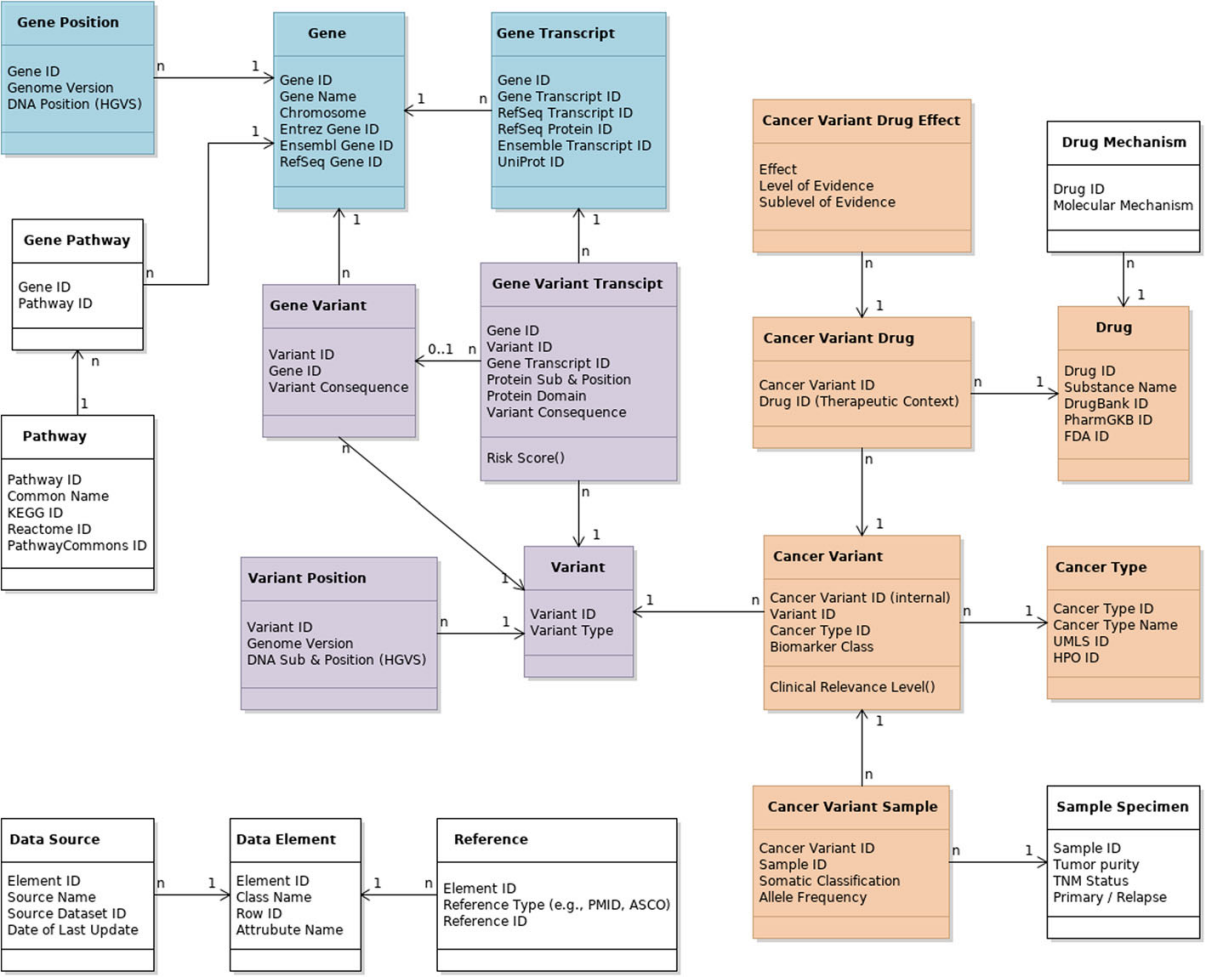
The relational class model to represent minimum variant level data (MVLD) and possible extensions; colors correspond to Ritter et al. [7]: brown: somatic interpretive data; purple: allele interpretive data; blue: allele descriptive data; white: background data extending MVLD. Cardinalities of relationships indicated as follows: (A)1–n(B): one instance of (A) is associated with an arbitrary number of instances of (B); (A)0..1–n(B): no or one instance of (A) is associated with an arbitrary number of instances of (B)

We developed a relational data model for representation of variant level data for molecular oncology. We choose a relational data model both because it can accurately represent the complex interrelationships between the various types of entities to be represented, and because it provides clear instructions for implementation of such a model inside a relational database management system^1^. Such implementation is well understood and has excellent tool support - which is a highly relevant aspect for widespread applicability and adoption. Figure 2 shows the relational class diagram of the resulting data model as the basis for a VIS implementation. Table 1 gives an overview of the corresponding data types and example values for each attribute. SQL files of both schema and sample data are included in the Additional files 1 and 2.

**Table 1:** Overview of data types and value ranges for data elements covered by the core data model for minimum variant level data

Class	Attribute	Value range	Example
Allele descriptive			
Gene	Gene ID	Internal ID	G0002V5Z
	Gene name	HGNC gene symbols	KRAS
	Chromosome	1.. 22, X, Y	12
	Entrez gene ID	Entrez gene IDs	3845
	Ensembl gene ID	Ensembl gene IDs	ENSG00000133703
	RefSeq gene ID	RefSeq gene IDs	NG_007524
Gene transript	Gene ID	Internal ID	G0002V5Z
	Gene transcript ID	Internal ID	T0006OOW
	RefSeq transcript ID	RefSeq Transcript IDs	NM_033360
	RefSeq protein ID	RefSeq protein IDs	NP_203524
	Ensemble transcript ID	Ensemble transcript IDs	ENST00000256078
	UniProt ID	UniProt IDs	P01116
Gene position	Gene ID	Internal ID	G0002V5Z
	Genome version	Genome build IDs	GRCh37.p13
	DNA position	Genomic coordinate	12p12.1
Gene pathway	Gene ID	Internal ID	G0002V5Z
	Pathway ID	Internal ID	P003V724
Gene pathway	Pathway ID	Internal ID	P003V724
	Common name	Activation of RAS in B cells	
	Kegg ID	Kegg IDs	map04014
	Reactome ID	Reactome IDs	R-HSA-1169092
	PathwayCommons ID	PathwayCommons IDs	R-HSA-1169092
Allele interpretive			
Variant	Variant ID	Internal ID	V0000LBB
	Variant type	“Single nucleotide variant (SNV)”, “multinucleotide variant (MNV)”, “insertion (INS)”, “deletion (DEL)”	SNV
Variant position	Variant ID	Internal ID	V0000LBB
	Genome version	Genome build IDs	GRCh37.p13
	DNA sub. & position	HGVS genomic coordinate	NC_000012.11:g.25398284C >G
Gene variant	Gene ID	Internal ID	G0002V5Z
	Variant ID	Internal ID	V0000LBB
	Variant consequence	“Non-sense”, “missense”, “silent”, “frame shift”, “in-frame”, “3UTR”, “5UTR”, “splice”, “splice-region”, “intronic”, “upstream”, “downstream”	missense
Gene variant transcript	Gene ID	Internal ID	G0002V5Z
	Variant ID	Internal ID	V0000LBB
	Gene transcript ID	Internal ID	T0006OOW
	Protein sub. & Position	HGVS formatted variants	NM_033360.3(KRAS):c.35G >C (p.Gly12Ala)
	Protein domain	Descriptive name of protein domain	Small GTP-binding protein domain
	Variant consequence	“Expression”, “amplification”, “deletion”, “fusion”, “loss of function”, “missense”	missense
	Risk score	FATHMM, SIFT, PolyPhen	0.98468, 0, 0.97
Somatic interpretive			
Cancer type	Cancer type ID	Internal ID	C000WQFL
	Cancer type name	NCI thesaurus | Oncotree IDs	Colorectal cancer
	UMLS ID	UMLS concept IDs	C1527249
	HPO ID	HPO concept IDs	HP:0003003
Cancer variant	Cancer variant ID	Internal ID	CV00XBQW
	Variant ID	Internal ID	V0000LBB
	Cancer type ID	Internal ID	C000WQFL
	Biomarker class	“Diagnostic”, “prognostic”, “predictive”, “predisposing”, “pharmacogenomic”	predictive
	Clinical relevance level()	“Tier 1”, “Tier 2”, “Tier 3” [8]	Tier 2
Cancer variant sample	Cancer variant ID	Internal ID	CV00XBQW
	Sample ID	Internal ID	SXBQW0A7
	Somatic classification	“Confirmed somatic”, “confirmed germline”, “unknown”	somatic
	Allele frequency	Allele frequency in global population	0.00001647
Sample specimen	Sample ID	Internal ID	SXBQW0A7
	Tumor purity	Ratio	0.763
	TNM status	TNM values	T2N1M1
	Primary / relapse	Primary || relapse	primary
Cancer variant drug	Cancer variant ID	Internal ID	CV00XBQW
	Drug ID	Internal ID	D00000Z9
Cancer variant drug effect	Cancer variant ID	Internal ID	CV00XBQW
	Drug ID	Internal ID	D00000Z9
	Effect	“Resistant”, “responsive”, “non-responsive”, “sensitive”, “reduced sensitivity”, “other”	Resistance or non-response
	Level of evidence	see Table 6	C
	Sublevel of evidence	see Table 6	3A
Drug	Drug ID	Internal ID	D00000Z9
	Substance name	FDA approved | DrugBank substance names	Panitumumab
	DrugBank ID	DrugBank IDs	DB01269
	PharmGKB ID	PharmGKB IDs	PA162373091
	FDA ID	FDA IDs	125147
Drug mechanism	Drug ID	Internal ID	D00000Z9
	Molecular mechanism	Description	Binds to the epidermal growth factor receptor (EGFR) on both normal and tumor cells[...]

Example data for evidence recording is given in Additional file 3

The data model is compliant with the requirements postulated in [7, 8], but also has several features not foreseen therein. In particular, it (1) acknowledges the fact that for many types of variant-associated information multiple and potentially diverging evidences exist, (2) allows the assignment of variants to transcripts (instead of only to genes) and thus splice variants, and (3) adds additional capacity for inclusion of clinically highly relevant background data such as prevalence and context of variants in public (or in-house) patient cohorts (e.g., The Cancer Genome Atlas (TCGA) [40] or the Exome Aggregation Consortium (ExAC) [41]).

In the following, we discuss the model’s elements in detail and explain our motivation for the concrete modeling constructs used; further considerations regarding linking of data elements to original evidences and extensibility of the model can be found in Additional file 3.

#### Entities, relationships, cardinalities

Following the classification used by Ritter et al. [7], data elements regarding cancer types, cancer samples, and drugs fall into the category *somatic interpretive*. Gene variants are described by the *allele interpretive*, and genes by the *allele descriptive* part of the schema.

##### Somatic interpretive data

The central entity of interest is the *Cancer Variant* representing a specific genomic variant in a specific cancer type. It is important to note that in the context of disease treatment and especially cancer therapy, the variant itself has to be directly associated with the respective cancer type to be fully interpretable. For instance, while a drug specifically targeting a certain gene transcript or even variant may be proven and approved for therapy in one particular cancer type, neither its approval, nor its efficacy may be assumed for other cancer types. An example of such a case is Vemurafenib, targeting BRAF V600 mutations in different non-melanoma cancers with great differences in observed response rates [42]. Furthermore, a particular *Biomarker Class* and a certain *Clinical Relevance* can only be assigned to a variant in the context of a specific cancer type. Note that we extend the set of biomarker classes suggested by MVLD (*diagnostic, prognostic, predictive*) with the additional classes *predisposing* and *pharmacogenomic* [43].

Both entities, *Cancer* and *Variant*, are referenced from this central joint *Cancer Variant* representation. Reflected by the cardinalities annotated to the referential links in Fig. 2, a single variant may be found in any given cancer type and, conversely, a given cancer type may encounter numerous single variants. The *Somatic Classification* to discern somatic from germline variants, and the measured *Allele Frequency* are attributes of a variant in a specific cancer sample, i.e., a particular *Cancer Variant Sample*, and represented as such. Extending MVLD, a corresponding *Sample Specimen* covers multiple such *Cancer Variant Samples* - and thus allows (re)identification of mutation profiles found in individual cancer type specimens. Such identification of concrete specimens is especially important when including data from large scale genetic characterization projects, such as TCGA [40] or 1000 genomes [44], but also for representing (cancer) cell lines. *Sample Specimen* data may also be characterized by clinical properties of the specimen and sample quality which is often a relevant criterion when judging and interpreting cancer variants in clinical practice. Such properties include *Tumor Purity*, *TNM* status of the specimen, or whether the specimen was taken from a tumor’s *Primary Manifestation* or a *Relapse*.

Associated with each single *Cancer Variant*, several *Cancer Variant Drugs* may have been found to have a certain *Effect* on the respective *Cancer* and *Variant* with a certain *Level of Evidence*. Different sources of information (e.g., different studies reporting a drug’s effect on a given cancer for a certain variant) may provide information at different levels of evidence, and may even describe different effects. The data model is tailored to accurately mirror such differences as *Cancer Variant Drug Effects*. How different, possibly contradicting reports are to be interpreted for clinical applicability is left to the clinical expert^2^.

Since, in turn, a given drug often has different effects, and even more often different levels of evidence depending on the respective cancer type and variant, generic information about the *Drug* itself is referenced from the cancer variant specific class. This may (extending MVLD) include additional information such as the *Molecular Mechanism* through which a drug exerts its effect.

##### Allele interpretive data

Information regarding the *Variant* itself falls into one of two classes: Data that depends on the reference genome used when mapping the variant, and data that is independent of it. The former especially includes the specific *Variant Position* within the DNA of the *Genome Version* of the used reference, and the *DNA Substitution* found in the variant call. A given variant can have numerous instances of such positional information associated with it^3^.

Other Variant related data elements, such as the *Variant Type*, the *Variant Consequence*, and the *Protein Substitution and Position* are included in the reference-independent set of attributes. A further important piece of information is the *Protein Domain* affected by a variant, i.e., the functional substructure of the protein that is modified. These four attributes actually belong to different classes of elements (see Fig. 2): While the *Variant Type* is intrinsic to the variant itself, the *Variant Consequence* depends on the *Gene* affected by the variant and is consequently externalized to a *Gene Variant* class because a given variant at a given genetic location can affect multiple genes, incurring different *Variant Consequences*. Similarly, both *Protein Substitution and Position* and *Protein Domain* describe a *Gene Variant Transcript*, i.e., one of several possible transcripts of a mutated gene. Note that we further extend transcript level data with the inclusion of methods to calculate the *Risk Score* of a variant in a transcript.

Finally, to accomodate genetic alterations measured on the transcript level, we include a dedicated field to record the *Variant Consequence* for each single transcript and provide a direct link to associate the *Cancer Variant Transcript* with the *Variant* class itself - circumventing the necessity to provide gene level variant details for transcript level aberrations. This allows to directly include RNA Seq data in the model, where only the effect (i.e., the *Variant Consequence*) of a variant may have been measured, such as overexpression of a certain transcript, without evidence of exactly which genomic alteration is causal of the observed aberration.

##### Allele descriptive data

Each *Gene Variant* is associated with the *Gene* it affects, which in turn may host multiple variants. Next to its ID, a gene has a *Name*, a *Chromosome* it is located on, and may have multiple *Gene Transcripts* identified by *RefSeq Transcript ID* and *RefSeq Protein ID*. As with variants, positional information (*DNA Position*) about genes is externalized to reflect its dependence on the particular *Genome Version* of the reference build and the subsequent multiplicity of such *Gene Positions* for each *Gene*. Additional information can be linked. For instance, data about *Pathways* a gene is involved in, as sketched in Fig. 2, is especially helpful in cases where only little actionable evidence is available and the identification of pathway information at least provides a starting point to discuss treatment options. Note that each gene may be part of multiple pathways, and each pathway will be comprised of several genes/proteins.

#### Reference IDs and ontologies

We include attributes for a number of different standardized identification systems for each type of entity in the data model (extending MVLD), as listed in Table 2. For example, we not only identify transcripts by their RefSeq ID (as suggested by MVLD), but also include the *Ensemble Transcript ID* because it is more universally defined and applicable than its RefSeq counterparts: by definition, every gene has a canonical Ensemble Transcript ID, whereas RefSeq identifiers are assigned to transcripts after they have been identified and cataloged [21]. Features such as this one ease the mapping of elements from different data sources and allow identification of elements, especially when data is not per se provided by means of standard ontologies and controlled vocabularies.

**Table 2:** Identifiers included in data model for cross-source entity identification

Entity type	Primary ID source	Further ID sources
Gene	Entrez gene	Ensembl, RefSeq
Transcripts	RefSeq	Ensembl, UniProt
Disease names	Disease ontology	UMLS, human phenotype ontology (HPO)
Drugs	DrugBank	PharmGKB, FDA
Pathways	KEGG	Gene ontology, PathwayCommons, Reactome

### Integration of public databases into the VIS model

The data model presented in the previous section is capable of accurately representing variant-level data as required for supporting clinical decision-making in precision oncology. In this section, we first discuss important sources of such data with respect to their usefulness for providing variant data to a VIS. Next, we present strategies for actually performing their technical and semantic integration.

**Fig. 3:**
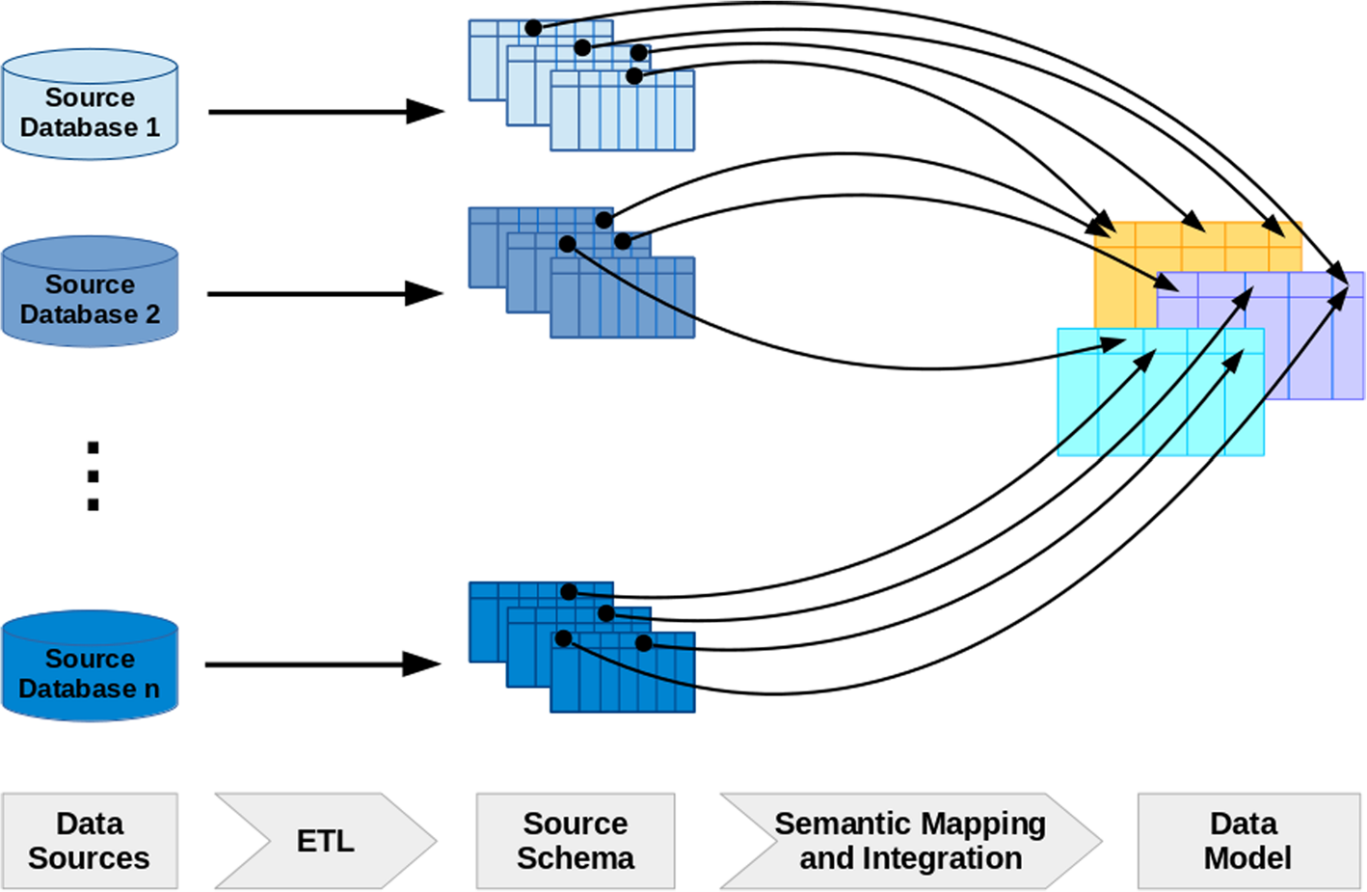
Overview of data integration: source databases are processed by extract/transform/load (ETL) scripts which generate source specific table spaces within the local database. From these, the relevant elements are semantically mapped to and loaded into the core data model

An overview of such an integration process is shown in Fig. 3. It consists of multiple steps: First, relevant data sources, i.e., high-quality variant level databases, need to be selected and understood. Next, these databases must be downloaded and integrated technically into the VIS, which typically leaves the data itself unchanged but makes it available for specialized database programming languages such as SQL. In the third step, the actual semantic integration of data must be performed to transform the source data into the target model. We discuss each of these steps in detail in the following sections.

#### Important public variant-level databases

Table 3 compares several important public cancer variant databases concerning their coverage of data represented in our data model. It is apparent that no single database provides all elements. In some cases the respective information is provided indirectly by linking to another data source within which the data can be found. From a technical perspective, most sources provide their data as compressed flat files in either CSV or TSV format. Those source files can be accessed via FTP or HTTP(s) and in some cases an API interface is provided. The files range in size from less than a MB to multiple GB and number from one file up to 94 files per source. The majority of sources is updated at a three month interval, however, some are updated nightly. When using flat files to gather the information from each source, it is imperative to automatically control the structure of those files before import and compare them to earlier versions, since any unnoticed restructuring may result in unexpected conflicts.

**Table 3:** Overview of databases integrated in our current implementation of the data model indicating which type of data is provided (’+’) by each database

	COSMIC	ClinVar	CIViC	Ensembl	1000 genomes	OncoKB	DrugBank	ClinicalTrials.gov	DGIdb	Cancer gene census	reactome	ncbi	DoCM	ExAC	canSAR	TCGA	GeneView	KEGG
**Genome version**	+	+		+	+					+			+		∼	+		
**Gene**	+	+	+	+	+	+	∼	∼	+	+	+	+	+	+	+	+		
**Chromosome**	+	+	+	+	+					+			+	+	∼	+		
**DNA position**	+	+	+	+	+					+			+	+	∼	+		
**RefSeq transcript**			+	+		+						+			∼	+		
**RefSeq protein**		∼	+	+		+						+			∼	+		
**Somatic classification**	∼	+		+	+			∼		+					+	+		
**DNA sub & position**	+	+	+	+	+	∼							+	∼	∼	+		
**Protein sub & position**	+	+	+	+	+	+							+	∼	∼	+		
**Variant type**	+	+	+	+	+	+				+			+	+	+	+		
**Variant consequence**	+	+	+	+		+				+				+	+	+		
**PMIDs**	+	+	+			+	∼		+				+			∼	+	+
**Cancer type**	∼	+	+	+		∼		∼		+			+		+	+		+
**Biomarker class**			+			∼										+		
**Therapeutic context**			+	+		∼	∼	∼	+							+		
**Effect**			+	+			∼	∼								+		
**Level of evidence**	+		+	+		∼				+								
**Sublevel of evidence**			+			∼												
**Pathway**							∼	∼			+				+			+
**Drug**			+				+	∼	+									
**Clinical significance**			+				+											
**Molecular mechanism**							+											
**Fully integrated**	+	+	+	+	+	+	+	+	+	+	+	+	+	+				
**Specifically linked**															+	+	+	+

In some cases the information is provided only in part (’ ∼’), meaning that either references to other sources are provided which hold the needed information or that the information is provided only for a subset of entities

#### Architecture for a data integration pipeline

Integrating data from a multitude of regularly updated sources requires a highly automated approach without manual intervention: scripted pipelines have to be created for each of the various sources to download the data, and to parse and transform it into a format which can then be loaded into the VIS [45]. Ideally, these pipelines should be robust to changes in the individual data models employed by the source databases, as these data models also evolve over time. One strategy to ensure such robustness is to decouple technical integration from the semantic integration required for actually making joint use of the integrated data. To this end, it is advisable to partition the table space within the target database to include separate data tables for each source that are consistent with the source schema, i.e., the relational layout of the data within the source database. The target model is filled from these tables in a subsequent step. This approach not only reduces error rates in physical integration, but also has the benefit of incorporating comprehensive, source specific data into the target database that may not be included in MVLD (and would require unreasonable upfront cost and effort to integrate into a global schema), but is readily available for further in depth analysis of data elements source by source.

#### Semantic data integration

The step of semantic integration moves and transforms each relevant data element from each of the sources’ local schemata into the VIS model. It is divided into two sub-problems [46]: (1) Mapping of schema elements, i.e., determining which elements of a source should be copied into which part of the target schema, and (2) mapping of data instances, i.e., determining how data values must be transformed to achieve a consistent representation. Both of these sub-problems have to cope with semantic heterogeneity between the elements considered. For schema elements, this occurs, for instance, when the information about the name of a given tumor in a data source is stored in an attribute called “cancer_entity”, while it is stored in an attribute “cancer_type” in the target schema. Such conflicts have to be detected and bridged. As schemas are typically small (a few dozen elements), resolution of conflicts at the schema level is usually performed manually using general data management tools. In contrast, conflicts at the instance level, such as two variants having different names, have to be resolved programmatically as millions of values are concerned. In the following, we discuss the most relevant data elements affected by such issues, and how appropriate solutions can be achieved.

##### Variant identification

Different data sources identify variants using a variety of namespaces. This results in ambiguities when mapping data to a particular variant. If provided, information such as chromosome position, assembly and range can be used to link the information of different sources to a variant. The HGVS (Human Genome Variation Society) notation [47], combining those pieces of information into a standardized string format, can be used to universally identify variants on various different genetic levels including the genomic location (g), coding DNA (c), RNA (r), protein (p), mitochondria (p) or non-coding DNA (n) location. However, even in its complete form, the HGVS identifier can be ambiguous, as the example in Table 4 shows. It is therefore advisable to only use a single designated authoritative source for the reference string. The genomic location (g) should be preferred over the more specific locational designators within HGVS, since it is subject to the underlying assembly only. A more detailed account of the considerations underpinning this recommendation can be found in Additional file 3.

**Table 4:** Ambiguities using HGVS nomenclature arising from overlapping genes and different sources, by the example of variant rs121913529

Ensembl HGVS	dbSNP HGVS	Associated
		gene
NC_000012.11:g.25398284C>G		KRAS
ENST00000256078.4:c.35G >C	NM_033360.3:c.35G >C	KRAS-004
ENSP00000256078.4:p.Gly12Ala	NP_203524.1:p.Gly12Ala	KRAS-004
ENST00000311936.3:c.35G >C	NM_004985.4:c.35G >C	KRAS-001
ENSP00000308495.3:p.Gly12Ala	NP_004976.2:p.Gly12Ala	KRAS-001
ENST00000556131.1:c.35G >C		KRAS-002
ENSP00000451856.1:p.Gly12Ala		KRAS-002
ENST00000557334.1:c.35G >C		KRAS-003
ENSP00000452512.1:p.Gly12Ala		KRAS-003

##### Molecular risk scores

An essential piece of information for assessing the clinical relevance of a given variant is its impact at the molecular level. Such information is contained in many variant-level databases and must be integrated into the VIS for inspection by the treating oncologists. In most cases, this information originally was computed by algorithms like SIFT [48], PolyPhen [49], and ClinGen [50]. These algorithms analyze signals such as the change of an amino acid and the corresponding changes of polarity or acidity in the protein, the creation of a stop or a start codon, or the provocation of a frame shift, to assess the functional impact of variants. However, the results of these programs are frequently inconsistent, as shown in Table 5.

**Table 5:** Assessment of the effect of different variants as provided by the SIFT and PolyPhen algorithms showing disagreement: While for variants one and two only one program calculates a score resembling the (true) clinical findings, variant three is corroborated by both programs and agrees with the clinical findings

rsID	SIFT	PolyPhen	Clinical evidence
rs104894359	0	0.361	Pathogenic
rs121913529	0	1	Pathogenic
rs1137282	0.85	0.012	Benign
Legend:			
SIFT:	0 (deleterious) - 1 (tolerated)		
PolyPhen:	0 (benign) - 1 (probably damaging)		

It is therefore recommended to use two or more independent algorithms to calculate risk scores for variants. If the assessments are integrated from a public data sources, care must be taken to check which programs were used to produce them, to be able to judge if these are independent evidences. During integration, all assessments should be kept and eventually be presented to the user.

##### Evidence levels

An essential piece of information for variant assessment are associations between a given variant, a tumor entity, and a drug. Such associations can be supported by different levels of evidence, ranging from FDA approved links to predictions based on in-silico analysis only. Databases storing such associations typically annotate them with distinct evidence levels which quantify the strength of the association; similarly, community standards sometimes define such levels. However, the scales and definitions used in practice are highly heterogeneous. In Table 6, definitions for evidence levels from Ritter et al. [7], CIViC [2], OncoKB [36], Meric-Berstam et al. [37], and Andre et al. [38] are compared. While all sources show a strong similarity in the definitions on the macro-level, the depth of the sub-leveling differs: Ritter et al. propose the usage of four tiers, CIViC defines five evidence levels and Andre et al. [38] suggest ten distinct levels. Mapping the sub-level of a given source to the corresponding macro-level is straight forward, however the sub-leveling of a given macro-level is challenging, since in depth literature research is required. Resolving such situations requires an experienced clinician. Again, a VIS should store each evidence level provided by individual data sources together with a detailed account of which source the respective entry was retrieved from.

**Table 6:** Evidence levels as defined by different sources

Evidence level	CIViC	[7]	Evidence level 2	Oncokb.org	Pct.mdanderson.org (PMID: 25863335)	Andre et. al. (PMID: 25344359)	Proposed
A / Tier 1	Validated association - proven/consensus association in human medicine	Alteration has matching FDA approved or NCCN recommended therapy	1A	FDA-approved biomarker and drug in this indication	Drug is FDA-approved for the same tumor type harboring a specific biomarker	Molecular alteration validated in several robust early phase trials or at least one phase III randomized trials. Alteration validated in the disease under consideration, targeted therapies have shown to be uneffective in patients who are lacking the genomic alteration	Drug is FDA-approved for the same tumor type harboring a specific biomarker
			1B		An adequately-powered, prospective study with biomarker selection/stratification, or a meta-analysus/overview demonstrates a biomarker predicts tumor response to a drug or that the drug is clinically effective in a biomarker-selected cohort in the same tumor type.	Molecular alteration validated in several robust early phase trials or at least one phase III randomized trials. No evidence that the therapy does not work in the absence of the molecular alteration	An adequately-powered, prospective study with biomarker selection/stratification, or a meta-analysus/overview demonstrates a biomarker predicts tumor response to a drug or that the drug is clinically effective in a biomarker-selected cohort in the same tumor type.
			1C			Molecular alteration validated in several robust early phase trials or at least one phase III randomized trials. Level I molecular alteration, but not in the disease under consideration	
B	Clinical evidence - clinical trial or other primary patient data supports association		2A	Standard-of-care biomarker and drug in this indication but not FDA-approved	Large-scale retrospective study demonstrates a biomarker is associated with tumor response to the drug in the same tumor type. This could be a prospective trial where biomarker study is the secondary objective, or an adequately powered retrospective cohort study or a case-control study	Efficacy of targeting molecular alteration suggested in single and underpowered phase I/II trials. Alteration validated in the disease under consideration, targeted therapies have shown to be ineffective in patients who are lacking the genomic alteration	Large-scale retrospective study demonstrates a biomarker is associated with tumor response to the drug in the same tumor type. This could be a prospective trial where biomarker study is the secondary objective, or an adequately powered retrospective cohort study or a case-control study
			2B	FDA-approved biomarker and drug in another indication, but not FDA or NCCN compendium-listed for this indication	Clinical data that the biomarker predicts tumor response to drug in a different tumor type	Efficacy of targeting molecular alteration suggested in single and underpowered phase I/II trials. No evidence that the therapy does not work in the absence of the molecular alteration	Clinical data (analogue 1A-2A) that the biomarker predicts tumor response to drug in a different tumor type
			2C			Efficacy of targeting molecular alteration suggested in single and underpowered phase I/II trials. Level I molecular alteration, but not in the disease under consideration or anecdotal evidence of response to targeting molecular alteration in single patient case reports	Single unusual responder (or case study) show a biomarker is associated with response to drug, supported by scientific rationales
C / Tier 2	Case study - individual case reports from clinical journals	Alteration has matching therapy based on evidence from clinical trials, case reports, or exceptional responders	3A	Clinical evidence links biomarker to drug response in this indication but neither biomarker or drug are FDA-approved or NCCN compendium-listed	Single unusual responder (or case studies) show a biomarker is associated with response to drug, supported by scientific rationales	Target suggested by preclinical studies. Preclinical studies include human samples, cell lines and animal models	Preclinical data (in vitro or in vivo models and functional genomics) demonstrates that a biomarker predicts response of cells to drug treatment in the same tumor type
D / Tier 3	Preclinical evidence - in vivo or in vitro models support association	Alteration predicts for response or resistance to therapy based on evidence from pre-clinical data (in vitro or in vivo models)	3B	Clinical evidence links biomarker to drug response in another indication but neither biomarker or drug are FDA-approved or NCCN compendium-listed	Preclinical data (in vitro or in vivo models and functional genomics) demonstrates that a biomarker predicts response of cells to drug treatment	Target suggested by preclinical studies. Preclinical studies that lack either cell lines or animal models	Preclinical data (in vitro or in vivo models and functional genomics) demonstrates that a biomarker predicts response of cells to drug treatment in a different tumor type
E / Tier 4	Inferential association - indirect evidence	Alteration is a putative oncogenic driver based on functional activation of a pathway	4A	Preclinical evidence associates this biomarker to drug response, where the biomarker and drug are NOT FDA-approved or NCCN compendium-listed		Target predicted but lack of clinical or preclinical data. Genomic alteration is a known cancer-related gene	Inferential association between biomarker and treatment response.
			4B			Target predicted but lack of clinical or preclinical data. Genomic alteration is not known as cancer-related gene	

## Discussion

We introduced an operational data model for implementing a VIS as a fundamental cornerstone of any evidence-based and genome-focused approach to precision oncology. Building on previously proposed standards regarding the set of relevant information and data elements, our model properly reflects biological, medical, and biotechnological interdependencies between all relevant entities. Furthermore, the model addresses a number of secondary issues regarding these data elements. In particular, it implements a rigorous representation of underlying original evidences, the representation of factual inconsistencies and heterogeneities between different, equally important data sources, and defined hooks for extensions to cater for future changes in our understanding of clinically relevant variant data. We proposed an architecture for populating this data model with data from databases of structured bio-oncological knowledge, describing a robust, multi-step integration process, and targeting practically viable solutions for challenges posed by both physical and semantic integration.

Nevertheless, our model still has a number of limitations which we discuss in the following sections.

***Offering up-to-date information*** Considering the pace at which new research results emerge in the fields of oncology and molecular genetics, the currency of information is a factor of great importance in oncological practice. Clearly, clinical decisions always should be derived from the most current data available. Obtaining the most up-to-date information, however, is difficult for many reasons. Firstly, relevant results take some time to appear in the (peer-reviewed) literature. Database curation incurs a further delay, as publications first have to be picked and analyzed by the curators before their structurized content becomes part of the database [6]. A third obstacle is the fact that relevant data is dispersed over multiple sources, requiring substantial time to be searched.

A VIS can alleviate only the third of these issues, by providing a single point of access for data from multiple sources. On the other hand, it introduces a singular source of delays, because updates in the primary databases take time to be ingested into the VIS. Many databases offer web user interfaces, which always work on the most current state of the database, yet only periodically provide complete database releases, which are the data sets integrated into a VIS. Although being slightly outdated, using such database releases has the advantage of defining a stable reference, which is a prerequisite for reproducible decisions. Consider a clinician taking a therapy decision based on information found in a curated variant database. When this decision, at a later stage, must be defended, it is vital to be able to exactly reproduce the state of this variant database at the time of retrieval; this is only possible if the release of the database at the time of the decision is known (and was archived), whereas online searches are not guaranteed to be reproducible as the underlying database might have changed in the meantime and only few online databases support queries against specific past versions of their data.

***Maintaining a VIS database*** Technically, approaches which build integrated VIS by periodically integrating releases of primary databases are called materialized systems because they maintain a copy of every data element [51]. An alternative are so-called virtualized systems, which forward user requests to the primary databases and build the integrated answer on-the-fly [52]. Virtual integration systems thus do not maintain any database themselves; instead, they only translate queries. The model we put forward in this work is a materialized system, which offers a number of advantages over virtual systems: They can answer queries faster, data within the database can be changed and corrected locally, they are more stable and offer higher availability, they allow a broader range of possible queries over the integrated data, and they allow reproducible decisions when the integrated databases don’t support versioned queries themselves. At the down-side, the most critical factor in a materialized VIS is its update frequency. VIS update procedures must be fast, robust, and be able to exchange the content of an updated data source at any time, all of which is only possible if a high degree of automation has been achieved. These procedures must also care about synchronization issues, which may occur if the state of data sources differ; for instance, one data source might still reference a gene which has already been deprecated in a newer reference genome. In our proposed integration process, we ease handling of such issues by the separation of source-specific data partitions from the target data model and by the two-step integration procedure. Still, we are not aware of any practical and general solution to the problem of asynchronous updates; instead, developers must implement their own, source- and VIS-specific strategies using custom ETL code.

***Exposing conflicting data*** Very often, different data sources provide diverging information regarding a variant’s impact in a specific tumor type or for the effect a drug has on a given variant. This fact creates a fundamental dilemma for any VIS based on data integration: Should all diverging information be kept and presented as such to clinicians, or should the VIS implement guidelines and rule sets for cleansing and aggregation of such issues upon data integration? The former strategy allows users to obtain a comprehensive and unbiased overview of existing data, and enables her to perform her own assessment. On the other hand, it also delegates responsibility for each decision to the individual user, and might be considered as creating information overload rather than efficiently supporting an informed decision. With the proposed data model, designed for explicitly tracking the lineage of single data elements, we currently recommend to use the former strategy because it provides maximum information – but it also expects a very knowledgeable user. To this end, current work to automate such aggregation in a clinically sound manner or privide guidance to clinical judgement is ongoing [46, 53, 54]. In the future, inclusion of such tools may become a viable option.

***Clinical relevance levels*** One particular issue in clinical oncology is the *Clinical Relevance Level* of a given cancer variant, which depends on a number of factors including available therapeutic drugs, the respective drug’s effect, or the biomarker class of the variant itself [8]. Any finding regarding any of these attributes itself is associated to an evidence level, roughly reflecting the trust in the truth of the finding. The Clinical Relevance Level is not included in our proposed data model as a regular attribute but rather as a user defined function which will produce the corresponding value from the respective input values at request time. While it may seem desirable to pre-compute such values, it must be kept in mind that many of the information such complex assessments are based on change over time. For instance, FDA approval of a drug for a given cancer type will lead to a change in its evidence level, which in turn will affect the respective variant’s relevance for clinical treatment. Thus, any system with precomputed clinical relevance levels must revisit and repeat these computations whenever their information sources have changed.

***Extensibility beyond cancer*** Arguably, oncology currently is one of the most active and actionable fields of variant related therapy. However, variants are also playing an increasingly important role in many other disease areas, such as immunology or genetics. While the data model proposed here specifically targets variant information in cancer, only few changes are required to allow its application also for other diseases. In particular, several relation names would be changed from Cancer to Disease (e.g., *Cancer Variant* to *Disease Variant*), one must add attributes to specify the specific *Disease Type* for each *Disease Variant*, and further, disease type specific information, must be modeled in additional tables, similar to how cancer specific information is currently represented by *Cancer Type*. With these changes, we believe that our proposed model is also applicable for representing variant level data in diseases outside the oncological spectrum.

***Beyond variants in coding regions*** The MVLD, on which our model is built, only covers variants in coding regions characterized by directly observable changes on the nucleotide level. In oncological practice, however, other types of genetic alterations play an increasingly important role. For instance, RNA sequencing detects changes at the transcriptome level, indicating that a gene (or rather one of its transcripts) is expressed, amplified, deleted, fused, etc. Such observations may form important, genetically determined biomarkers for diagnosis and decision, yet their direct cause, such as a mutation in a transcription factor binding side or a large-scale duplication of chromosomal regions, often remains hidden. We have shown how such alterations are included in our model by extending the vocabulary for *Variant Type*, by tracking *Variant Consequences* at the transcript level (see Table 1), and by providing relations for transcriptional alterations without a known association to a genomic variant. However, making transcriptome aberrations first class citizens in our data model would require extending the HGVS nomenclature itself, which is beyond the scope of our work.

Analogously, alterations of non-coding regions affecting, for example, miRNA, promoter regions, chromatin structure, or epigenetic factors, are currently not adequately represented in the data model. Furthermore, the model cannot capture the combined effects of multiple variants in the same sample, where the essential information could be the influence on a pathway and not at a single gene level. The foreseeable necessity of including such data into a VIS data model underlines the advantages of designing the data model to accurately reflect the true biological relationships between the involved entities. Only then can additional relationships be seamlessly integrated into the existing model.

## Conclusions

Variant Information Systems (VIS) are becoming a fundamental necessity for scaling clinical availability of comprehensive variant information in precision oncology. However, the informed selection of data elements to include in a VIS, the accurate design of the data model to hold this data, and the integration of existing data into that data model pose a number of challenges both from a clinical, and from a technical perspective. To this end, this paper provides a threefold contribution: Firstly, we performed a detailed analysis of data requirements for a VIS, incorporating existing and emerging community standards, own experience with both precision oncology and biomedical information systems, and the technical reality of existing sources of variant related data. Taking from this analysis, secondly, we designed and presented an implementation-ready data model to host a comprehensive set of data elements necessary for clinical utility and technical compatibility of variant information. And thirdly, we gave an instructional description of methods to acquire and merge data from a large number of heterogeneous public data sources to fill the model, together with a critical discussion of the technical and conceptual challenges such integration comes with. We believe that both the anlaysis and the solutions provided here will be highly instrumental to the community for the creation of comprehensive Variant Information Systems.

## Additional files

Additional file 1 SQL schema file implementing the data model presented in this paper. (SQL 5 kb)

Additional file 2 SQL data file for filling the schema with sample data as listed in Table 1. (SQL 4 kb)

Additional file 3 Additional considerations regarding (a) how the data model presented in “Results” section recognizes the design principles of extensibility and data lineage introduced in “Methods” section; and (b) variant identification for sematic integration when aggregating data from multiple source databases. (PDF 95 kb)

## Abbreviations

API: Application programming interface
CSV: Comma separated values
DO: Disease ontology
ETL: Extract, transform, load
FTP: File transfer protocol
GB: Gigabyte
HGVS: Human genome variation society
HPO: Human phenotype ontology
HTTP: Hyper text transfer protocol
KEGG: Kyoto encyclopedia of genes and genomes
MB: Megabyte
MVLD: Minimum variant level data
NEN: Named entity normalization
NER: Named entity recognition
RefSeq: NCBI reference sequence database
TSV: Tabular separated values
UMLS: Uniform medical language system
VIS: Variant information system

## Footnotes

1 As opposed to Entity Relationship (ER) diagrams

2 By clinical expert we mean any person using a VIS in patient care. Note, however, that such users typically do not directly access a database system but use intermediate applications. These applications may, again, perform certain data filtering or aggregation, implementing, for instance, organization-wide standards.

3 We discuss practical consequences of this multiplicity for variant level data integration in the next section.
