# Supplementary material for: Variant information systems for precision oncology
Source: BMC Med Inform Decis Mak. 2018 Nov 21;18:107. doi: 10.1186/s12911-018-0665-z (PMC6249891; doi:10.1186/s12911-018-0665-z)
Supplement: Supplementary file 3 — Additional considerations regarding (a) how the data model presented in “Results” section recognizes the design principles of extensibility and data lineage introduced in “Methods” section; and (b) variant identification for sematic integration when aggregating data from multiple source databases. (PDF 95 kb) [file 12911_2018_665_MOESM3_ESM.pdf]

Supplementary Material 1

Additional Results Regarding the Data Model

Adding to the description of our data model given in Section *Results, Data Model*, we here describe how the design principles of *linking data to evidence* and *extensibility* are represented in our data model.

Linking Data to Evidence

To allow for the assignment of scientific references to each element of the data model, a sub-schema is employed which allows to address each *Data Element* based on its *Class Name* (e.g., Variant), the *Row ID* of the respective data instance, and the *Attribute Name* within the class which holds the data element referred to in the reference. Following our considerations regarding varying sources of reference, the *Reference* associated to the Data Element via its (artificial) *Element ID* has both a *Reference Type* (e.g., PubMed or ASCO) and a *Reference ID*, indicating the concrete, say, PMID.

Next to this scientific evidence, the source a data element has been derived from needs to be recorded. The *Data Source* is described by its *Source Name*, (e.g., COSMIC or ClinVar), its *Source Dataset ID*, and the date of the last update. Following the example given in Table 1, the attribute *Effect* (holding the value "Resistance or Non-Response") for the drug Panitumumab in metastatic colorectal cancer in the presence of the KRAS G12A mutation recorded in class *Cancer Variant Drug Effect* has an associated reference of type *PubMed* with reference id *18316791*, and the data was retrieved from the data source with name *CIViC*.

Extensibility

In the description of the relational data model, we highlighted several possible extensions of the MVLD standard proposal we see necessary even today - showcasing how the extensibility of the model is facilitated by its modular design. Extensions of the model generally fall into 3 different categories. Extending *existing entities with new properties* is most straightforward and only requires the addition of the respective attribute to the corresponding entity class. For instance, to add a new type of identifier for genes, a new field with a suitable data type would be added to the *Gene* class, as done in our data model when extending the set of attributes proposed by MVLD for describing a gene by the *Ensemble Gene ID.*

Extending *existing vocabularies and terminologies*, as we have done for the nomenclature used to describe *Biomarker Classes* by adding *predisposing* and *pharmacogenomic* to the list of values covered, only requires minimal changes to the data model, as well. It may, however, require special attention regarding data stored within the model when existing terms are removed or refined by the extended vocabulary. In such cases, a multi-step approach may be necessary to first add new terms, possibly re-map data to the new terminology, and finally remove obsolete terms.

To extend the model with an *additional type of entity*, a new entity class and its relations to existing classes would be added. For example, to accurately represent the (potentially multiple) types of sequencing a *Cancer Variant Sample* was discovered by in a given *Sample Specimen*, a new entity class would be added, say *Sample Sequencing*, with relations to both of the aforementioned classes: 1 *Sample Specimen* to n *Sample Sequencings*, and n *Sample Sequencings* to m *Cancer Variant Samples.*

Additional Rationale within the Data Integration Process

We here extend on the considerations regarding cross-source variant identification, as outlined in *Results, Integration of Public Databases into the VIS Model.*

Variant Identification for Semantic Data Integration

Different data sources identify variants, genes, cancer types, and other types of entities using a variety of namespaces for each entity type (e.g., Ensemble, RefSeq, or Entrez for genes). To allow consistent identification and mapping of information from different data sources, our integrative data model maintains several namespaces for each type of

entity. Even when a variety of such identifiers are available, the greatest obstacle in integrating variant information from different sources still is the lack of a universally unique identifier for all variants which would allow the direct and unambiguous mapping of information from any given source to a particular variant. Most databases use an internal ID for variants. While internally these IDs may be unique, they do not directly relate to their respective counterparts in the context of multiple independent databases. Given certain information about a variant, such as chromosome position, range and assembly, it is possible to map variants among most sources. If the assembly is provided, the location can even be recalculated between different assemblies, but often this information is not provided or implied, and a variant first described on assembly x can not be mapped directly to information based on assembly y. Location information based on gene names, transcripts, chromosome bands or other annotated entities are subject to the same limitations.

In the absence of a unique identifier most authors use some form of the HGVS notation [1] to ensure the recognition of a given variant. However, in many cases this nomenclature is not a unique identifier either: Many publications and databases store variant information as, for instance, <HUGO>:c.[0-9]+[ATGC]>[ATGC], stating the name of the gene, the coding DNA position and the base exchange. Here the location is ambiguous, because the reference information is missing. Using a different assembly and annotation this variant might be at a different location. When using the HGVS notation the complete notation should be used, allowing for the addition of reference information: <Reference>(<HUGO>):c.[0-9]+[ATGC]>[ATGC].

Even if provided, transforming the reference string into useful information to utilize during integration takes a great deal of effort and the use of multiple cross reference tables. It is therefore advisable to only use a single designated authoritative source for the reference string to simplify the continued use of variant information.

Further complicating the mapping process, HGVS allows the use of different locations in the notation depending on the type of the underlying reference (genome g, coding DNA c, RNA r, protein p, mitochondria p, non-coding DNA n). While the genomic location is based solely on the underlying assembly, the coding DNA and protein locations are based mainly on the annotation, i.e., an assignment of transcriptional and translational outcomes to the respective variant. Table 4 shows the ambiguity that arises even between only two different data sources - each of which is using the complete HGVS notation and a distinct reference. The example also illustrates the problem arising from overlapping genes and transcripts. The single SNP listed in Table 4 has four distinct 'c' notations, four corresponding 'p' notations and one 'g' notation in Ensembl [2].

As such, location information is more or less stable or unique depending on the reference. In many applications, using the protein based notation to reflect the changes on the amino acid level, is the most useful way to represent a variant, and the one most commonly used in clinical practice. However, the ambiguities arising from overlapping entities, the usage of single or triple letter amino acid codes and the dependence on an annotation reduce the usefulness of this notation for identifying and storing variant information. Since, on the other hand, the genomic location is most straight forward, depends only on the assembly and can easily be transformed into the other location notations using the corresponding annotations (where available), it should be preferred as the least ambiguous notation when storing variant information.

References

[1] den Dunnen, J.T., Dalgleish, R., Maglott, D.R., Hart, R.K., Greenblatt, M.S., McGowan-Jordan, J., Roux, A.-F., Smith, T., Antonarakis, S.E., Taschner, P.E.: Hgvs recommendations for the description of sequence variants: 2016 update. Human mutation (2016)

[2] Yates, A., Akanni, W., Amode, M.R., Barrell, D., Billis, K., Carvalho-Silva, D., Cummins, C., Clapham, P., Fitzgerald, S., Gil, L., *et al.*: En-

sembl 2016. Nucleic acids research **44**(D1), 710–716 (2016)
